# The potential role of lily polysaccharide in mitigating radiation-induced pneumonitis via the gut-lung axis: a comprehensive review

**DOI:** 10.3389/fphar.2026.1863108

**Published:** 2026-06-29

**Authors:** Yuan Wang, Zhongli Zheng, Miaomiao Zhang, Jingru Zhao, Quanle Xu, Wenjun Wei, Jufang Wang, Nan Ding

**Affiliations:** 1 Institute of Modern Physics, Chinese Academy of Sciences, Lanzhou, China; 2 Key Laboratory of Space Radiobiology of Gansu Province, Lanzhou, China; 3 University of Chinese Academy of Sciences, Beijing, China; 4 Gansu Rehabilitation Center Hospital, Lanzhou, Gansu, China; 5 College of Life Sciences, Northwest A&F University, Yangling, Shaanxi, China

**Keywords:** cGAS-STING- NLRP3 signaling, gut microbiota, gut-lung axis, lily polysaccharide, radiation-induced pneumonitis

## Abstract

Radiation-induced pneumonitis (RIP) is a common and severe complication of thoracic irradiation, for which effective clinical treatments remain limited. Lily polysaccharide (LP), a natural polysaccharide derived from Lilium bulbs, exhibits various biological activities, including anti-inflammatory and tissue-protective effects. However, following oral administration, LP is poorly absorbed from the gastrointestinal tract and thus cannot directly target the lungs. Drawing on the gut-lung axis (GLA) theory, we hypothesize that oral LP may alleviate RIP by remotely inhibiting abnormal activation of the pulmonary cGAS-STING-NLRP3 signaling axis through modulation of the gut microbiota (GM) and its metabolites. Nevertheless, current evidence supporting this hypothesis remains largely indirect. Whether LP metabolites directly inhibit the pulmonary cGAS-STING-NLRP3 pathway, and whether such inhibition is causally linked to changes in the GM, require validation in RIP models. Therefore, this paper systematically reviews the molecular mechanisms underlying RIP, the multi-target pharmacological activities of LP, and the cross-organ regulatory network of the GLA. It also identifies key gaps in current research and provides a theoretical framework for future in-depth investigations, including fecal microbiota transplantation, germ-free animal models, structure-activity relationship analyses, and clinical safety assessments.

## 1 Introduction

Radiation-induced pneumonitis (RIP) is the early phase of radiation-induced lung injury (RILI), typically occurring 1–3 weeks post-irradiation (Dasgupta et al., 2023). Currently, RIP has emerged as a major complication that limits dosage escalation in radiotherapy and adversely affects patient quality of life and prognosis, with an incidence rate of 10%–30%. The acute phase is characterized by an obvious inflammatory response, and severe cases can progress to irreversible radiation-induced pulmonary fibrosis (RIPF), a potentially life-threatening condition (Hou et al., 2025; Wang et al., 2026). Classical understanding holds that RIP is directly triggered by an ionizing radiation-induced burst of reactive oxygen species (ROS), which initiates an acute inflammatory cascade (Zhang et al., 2025).

However, advances in our understanding of the cGAS-STING-NLRP3 axis have substantially deepened mechanistic insights into RIP. Radiation exposure induces mitochondrial dysfunction, leading to the leakage of mitochondrial DNA (mtDNA) into the cytoplasm, which in turn activates the cGAS-STING pathway (Peng et al., 2024; Zhang et al., 2023). Activation of this pathway promotes NLRP3 inflammasome assembly, resulting in pyroptosis and subsequent release of IL-1β and IL-18 (Zhang et al., 2025). Collectively, these events drive inflammatory response.

Despite these insights, current clinical management of RIP remains largely dependent on corticosteroids and antioxidants (Ruysscher et al., 2025; Sharma et al., 2022; Voruganti Maddali et al., 2024), which are associated with immunosuppression and hormone dependence and fail to prevent disease progression definitively. These limitations create an opportunity for the exploration of natural product-based interventions. Consequently, the development of safe, effective, and mechanistically well-defined preventive and therapeutic strategies has become a central focus in contemporary radiotherapy and pulmonary research.

Natural plant polysaccharides, a major class of biomacromolecules found in plant tissues such as roots and fruits, have shown promise for radioprotection owing to their low toxicity and multi-target properties (Ruiqing et al., 2025; Shi et al., 2026). An et al. (2025) identified the core bioactive metabolites in lily and demonstrated their anti-inflammatory, immunomodulatory, antioxidant, and tissue-protective activities. Lily polysaccharide (LP), a mixed polysaccharide complex extracted from the fleshy bulbs of Liliaceae plants, effectively scavenges ROS and alleviates pulmonary edema and inflammatory cell infiltration in lung injury models by inhibiting inflammatory signaling pathways such as NF-κB (Liu R. et al., 2025; Shi et al., 2026).

However, as macro-molecular hydrophilic heteropolysaccharides, natural polysaccharides upon oral administration are not directly absorbed from the gut into the bloodstream or lungs; instead, they reach the colon intact and are metabolized by the gut microbiota (GM) into oligosaccharides or short-chain fatty acids (SCFAs) (Ji et al., 2026; Li et al., 2017). Based on the pharmacokinetic profile of LP, we hypothesize that its anti-RIP effect is not mediated by direct pulmonary intervention but rather relies on gut microbial metabolism, operating through an indirect sequential gut-to-lung mechanism. Specifically, LP may exert prebiotic effects by restoring the integrity of the intestinal mucosal barrier and promoting the proliferation of beneficial bacteria, whereas metabolites derived from the GM can enter the systemic circulation and modulate immune responses in the lungs.

The gut-lung axis (GLA) theory posits that GM and their metabolites remotely regulate pulmonary immune homeostasis via the bloodstream and immune cell migration (Le Guern et al., 2023; Liu J. et al., 2025). Accordingly, we hypothesize that oral administration of LP alleviates RIP by modulating gut microbial composition and metabolic function, repairing the intestinal mucosal barrier, and thereby remotely suppressing excessive activation of the pulmonary cGAS-STING-NLRP3 axis via the GLA.

Therefore, this review aims to systematically evaluate the available evidence supporting this hypothesis, clearly differentiate direct from indirect evidence, and identify key research gaps, thereby offering novel insights for future mechanistic studies and clinical translation of LP in the prevention and treatment of RIP.

## 2 Multi-target protective mechanisms of LP against RIP

### 2.1 The mechanisms of RIP

**FIGURE 1:**
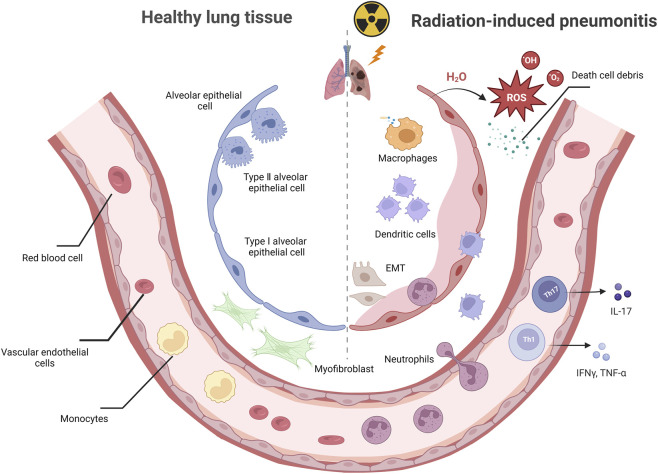
The mechanism of radiation-induced pneumonitis, Created in BioRender.com.

Traditional research suggests that RIP is characterized by apoptosis of type I alveolar epithelial cells, dysfunction of type II alveolar epithelial cells, and reduced pulmonary surfactant secretion, ultimately leading to the release of large numbers of inflammatory mediators into the alveoli and driving pulmonary inflammation (Feng et al., 2024; Yang et al., 2022). The pathological process of RIP involves ROS-mediated oxidative stress, neutrophil recruitment, monocyte activation into macrophages (Canfrán-Duque et al., 2023), antigen presentation by dendritic cells (DCs) (Bozward et al., 2025), and immune dysregulation of T and B cells (Cembellin-Prieto et al., 2025) (Figure 1). However, the clinical phenomenon of “severe early inflammation” in RIP cannot be fully explained by traditional linear models. Currently, the cGAS-STING pathway and NLRP3 inflammasome-mediated pyroptosis have been established as key molecular bridges linking radiation-induced injury to inflammatory responses, substantially advancing our theoretical understanding of the pathogenesis of RIP.

### 2.2 The cGAS-STING-NLRP3 axis: a potential driver of RIP

The cGAS-STING signaling axis serves as a critical bridge connecting radiation-induced DNA damage to acute inflammation. Hu et al. (2026) demonstrated that DNA double-strand breaks and the accumulation of cytoplasmic double-stranded DNA (dsDNA) in lung tissue following ionizing radiation activate the cGAS-STING axis in a time-dependent manner, inducing phosphorylation of TBK1 and IRF3. Upon activation, cGAS catalyzes the generation of cGAMP from ATP and GTP, triggering a cascade that significantly upregulates inflammatory gene expression and induces type I interferon production (Wang H. et al., 2025; Zhi et al., 2022). Importantly, STING promotes the assembly and activation of the NLRP3 inflammasome, which in turn activates caspase-1. Activated caspase-1 cleaves pro-IL-1β and pro-IL-18 to produce the mature inflammatory cytokines IL-1β and IL-18, and simultaneously cleaves gasdermin D (GSDMD) to release its pore-forming active N-terminal fragment (GSDMD-NT). This fragment oligomerizes to form membrane pores, triggering pyroptosis and the release of large amounts of inflammatory cytokines (Cao et al., 2024; Xiao et al., 2023; Zhang et al., 2022; Zhang et al., 2023). In mouse models, inhibition of STING or NLRP3 significantly relieves pulmonary inflammation (Cheng et al., 2022; Yang et al., 2024). Although no direct evidence currently shows that the cGAS-STING-NLRP3 axis drives RIP, we propose that targeting this axis may represent a key strategy for the prevention and treatment of RIP, thereby providing a clear molecular target for LP-based intervention studies.

### 2.3 Crosstalk between the cGAS-STING-NLRP3 axis and other related signaling pathways

The pathological progression of RIP is regulated by the synergistic interplay of multiple signaling pathways. In addition to the cGAS-STING-NLRP3 axis, pathways such as Nrf2, NF-κB, and MAPK also play critical roles, collectively forming a complex immune network (Xiong et al., 2021). For example, Nrf2 activation inhibits M1-type macrophage polarization while promoting M2-type polarization, and it exhibits crosstalk with NF-κB (Tao et al., 2021; Xiong et al., 2025). The compound HD exerts pulmonary protective effects by activating the Keap1/Nrf2/HO-1 pathway to alleviate oxidative stress while simultaneously inhibiting the MAPK/NF-κB pathway to reduce inflammation (Hu et al., 2023). Moreover, activation of NF-κB downstream of the cGAS-STING pathway induces NLRP3 inflammasome activation and promotes IL-1β secretion (Mun et al., 2025; Neufeldt et al., 2022; Zhong et al., 2024). Concurrently, the release of radiation-induced damage-associated molecular patterns (DAMPs), such as dsDNA, can activate both the TLR pathway and the cGAS-STING pathway. Additionally, the JAK/STAT pathway interacts with cGAS-STING, NF-κB, and NLRP3 signaling to regulate neuroinflammation (Shen et al., 2023). In the context of adaptive immunity, abnormal activation in RIP manifests as a Th17/Treg imbalance (Shen et al., 2026); however, the crosstalk between these adaptive immune components and innate immune pathways remains unclear and requires further investigation. Importantly, most current evidence on pathway crosstalk comes from non-RIP models or *in vitro* experiments. Therefore, the dynamic synergistic or antagonistic relationships among these pathways in the specific pathological context of RIP still require systematic validation.

### 2.4 Potential protective role of LP

**FIGURE 2:**
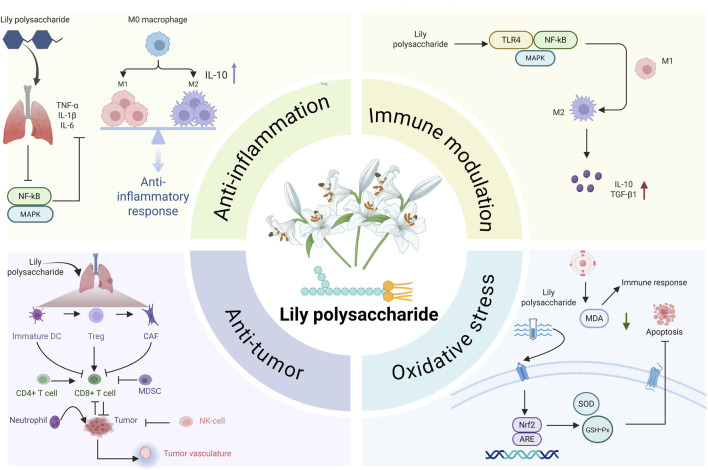
The biological activities of lily polysaccharides, Created in BioRender.com.

Among the bioactive constituents of lily, LP is one of the most significant, exhibiting a broad range of biological activities, including anti-inflammatory, antioxidant, immunomodulatory, and anti-tumor effects (Figure 2).

The extraction yield of polysaccharides has been reported to be 18.25% for Lanzhou lily, 10.43% for Lilium lancifolium, and 8.9%–12.8% for wild lily (Song et al., 2022) (Table 1). Furthermore, a separate study demonstrated that BHP-1 and LPR, two heteropolysaccharides derived from Lanzhou lily, are composed of glucose and mannose in molar ratios of 5.9:2 and 2.9:3.3, with molecular weights of 1.93 × 10^5^ Da and 5.12 × 10^4^ Da, respectively (Zhou, 2024). These characteristics reflect differences among lily varieties, and the abundant lily resources in Lanzhou provide a favorable material basis for our further investigation into the GM-dependent indirect mechanism of LP following oral administration.

**TABLE 1:** Comparison of structural characteristics and biological activities of polysaccharides from different lily varieties.

Species	Polysaccharide type	Molecular weight	Polysaccharide content	Biological activities	References
Lanzhou lily	α-(1→6)-D-glucan; heteropolysaccharides BHP-1, BHP-2	BHP-1: 7.26 kDa; BHP-2: 15.43 kDa	18.25%	Antioxidant (DPPH/·OH/ABTS), antimicrobial, immunomodulatory, antitumor	Shi et al. (2026), Song et al. (2022)
Lilium lancifolium	LP-1 (linear O-acetyl glucomannan); L005-B (a novel polysaccharide)	LP-1: 5.3 kDa; L005-B: 43.9 kDa	10.43%	LP-1: antioxidant, endothelial protection; L005-B: anti-pulmonary fibrosis	Liu R. et al. (2025), Song et al. (2022)
*Lilium davidii* Duch. ex Elwes	S-LP (subcritical water extraction); U-LP (ultrasound-assisted extraction)	S-LP: 245.0–645.0 kDa; U-LP: 2.4–287.0 kDa	7.92%∼11.48%	Antioxidant, immunomodulatory, anti-radiation, anti-inflammatory, antitumor, renal protection	Liu and Liao (2025)
Longya Baihe	Pectic polysaccharide WLBP-A3-c; glucomannan LBP; heteropolysaccharide LP	WLBP-A3-c: 59 kDa; LBP: 312 kDa; LP: 24.006 kDa	11.31%	Antioxidant, lipid-lowering, anti-inflammatory, immunomodulatory	Guo Y. et al. (2025), Li et al. (2024), Mowat and Agace (2014)

#### 2.4.1 Targeted regulatory mechanisms of anti-inflammatory and anti-fibrotic effects of LP

LP has significant research value for preventing and treating inflammation-related diseases, owing to its multi-stage intervention capability, multi-target effects, natural origin, and low toxicity. Polysaccharides derived from Lilium brownii var. Viridulum (Li et al., 2025a) suppress inflammation at its source by downregulating pro-inflammatory cytokines while upregulating anti-inflammatory cytokines. Moreover, synergistic interactions between polysaccharides and other lily components, such as steroidal saponins and polyphenols, enhance anti-inflammatory properties by using the combined effects of multiple bioactive compounds. This synergy overcomes the limitations of single-component approaches and opens new avenues for combined protective strategies using natural products. (Wang Y. F. et al., 2024).

Numerous studies have confirmed that LP significantly alleviates pulmonary fibrosis, as demonstrated by the L005-B glycan extracted from lily. This glycan reduces hydroxyproline (Hyp) levels in lung tissue and downregulates fibrotic markers, including alpha-smooth muscle actin (α-SMA) and type I collagen (Col-I) (Liu R. et al., 2025). In a bleomycin-induced mouse model of pulmonary fibrosis, BHP-1, a polysaccharide purified from the bulbs of Lanzhou lily, exhibited potent *in vivo* bioactivity, significantly improving increased lung indices, histopathological abnormalities, and collagen deposition (Chen et al., 2020). Furthermore, Wang et al. (2022) demonstrated that natural polysaccharides possess broad-spectrum anti-fibrotic activity, reducing fibrosis severity and injury across multiple organs (e.g., heart, lung, liver, kidney) by modulating signaling pathways such as TGF-β/Smad, HMGB1/TLR4, and cAMP/PKA. Together, these findings suggest that LP may exert dual anti-inflammatory and anti-fibrotic effects, providing an experimental basis for the prevention and treatment of RIP and RIPF.

#### 2.4.2 Synergistic mechanisms in targeted immune homeostasis regulation

LP acts as a novel immunomodulator that bidirectionally regulates radiation-induced immune dysregulation (Ruiqing et al., 2025). Studies show that LP-based nanoparticles synergize with curcumin to enhance the antigen-presenting capacity of macrophages (Shi et al., 2026). Furthermore, LP reverses radiation-induced immunosuppression by modulating endothelial phagocytic activity and specific humoral immunity, and its differential bioactivity correlates with the activation of Toll-like receptor (TLR) family members (Li et al., 2024; Wang et al., 2019). In addition, structural modifications, such as selenite conjugation, improve the affinity of LP for immune receptors (Hou et al., 2016).

#### 2.4.3 Protective mechanisms targeting oxidative stress

LP exhibits potent antioxidant activity (Lakshmanan et al., 2023), a property largely determined by the number and spatial arrangement of functional groups such as hydroxyl and carboxyl groups in its structure. These structural features enable LP to chelate metal ions and activate the Nrf2/ARE pathway, thereby promoting the *in vivo* synthesis of superoxide dismutase (SOD) and glutathione peroxidase (GSH-Px) (Gao et al., 2022; Hui et al., 2019). In terms of radioprotection, LP reduce malondialdehyde (MDA) levels, selectively inhibit radiation-induced apoptosis, enhance resistance to radiotherapy, and provide specific protection to immune organs such as the spleen (Guo Z. et al., 2025). Differences in the secondary metabolite profiles among lily species contribute significantly to their varying antioxidant capacities (Tang et al., 2021). The broad-spectrum antioxidant activity of multiple bioactive components in lily bulbs supports the screening and targeted breeding of LP strains with high bioactivity (Zhou et al., 2024). Moreover, a LP fragment exhibits significant radioprotective effects, promoting human umbilical vein endothelial cell proliferation and attenuating X-ray-induced DNA double-strand breaks, highlighting its potential as a natural antioxidant (Kang et al., 2022). Collectively, these findings provide an experimental basis for a deeper understanding of the radioprotective mechanisms of LP under complex pathological conditions.

#### 2.4.4 Regulatory mechanisms of targeted anti-tumor effects

Polysaccharides exert their anti-tumor effects primarily through immunomodulatory mechanisms (Zhang M. et al., 2025). Studies have shown that LP60-1, a water-soluble polysaccharide isolated from lily, demonstrates significant anti-tumor activity against MDA-MB-231, A549, HepG2, and MCF7 cells (Chen P. et al., 2025; Zhao et al., 2002). Moreover, LBPS-I, a side-chain glucan structure present in LP, inhibits the growth of B16 melanoma and Lewis lung carcinoma by activating immune cells (Zhao et al., 2002). The pharmacological activity of LP largely results from its interaction with various membrane receptors and its ability to trigger intrinsic and extrinsic apoptotic cascades, ultimately inducing tumor cell apoptosis (Guo et al., 2024). In addition, selenium nanoparticles synthesized using LP can arrest the cell cycle and induce apoptosis in A549 cells (Wei et al., 2024). Xylulose-5-phosphate, a bioactive compound derived from lily, enhances the cytotoxic activity of CD8^+^ T cells via xylulokinase; deficiency of xylulokinase impairs the infiltration and activation of CD8^+^ T cells in tumor metastasis, thereby mediating the observed anti-tumor effect (Shi et al., 2025). Collectively, these findings provide a theoretical foundation for the immunotherapy of metastatic cancer.

In addition to studies on LP and its active components, various natural plant polysaccharides also exhibit radio-protective, antioxidant, and immunomodulatory activities. Multi-perspective analyses indicate that these polysaccharides offer multi-target potential against inflammation and oxidative damage, pointing to future research directions on LP functions. Representative studies are summarized in Table 2.

**TABLE 2:** Bioactivities and mechanisms of plant polysaccharides in *in vitro* and *in vivo* models.

Study design	Model/Cell	Polysaccharide	Key findings	Mechanism	Limitations	Reference
*In vitro*	HaCaT cells	Lycium barbarum polysaccharide-glycoprotein (LBP)	① LBP enhanced the viability of irradiated HaCaT cells	Activates Nrf2 signaling; upregulates HO-1, NQO1, SLC7A11, and FTH1 to boost antioxidant capacity and suppress oxidative stress damage	The study did not validate the role of upstream regulatory molecules through *in vitro* experiments, nor did it compare the activity differences of LBP prepared using different concentrations or extraction methods	Dasgupta et al. (2023)
			② LBP suppressed radiation-induced oxidative stress			
	lung cancer cells	natural plant polysaccharides	① Inhibits lung cancer cell growth and suppresses tumor proliferation ② Enhances immune function ③ Regulates GM and improves therapeutic efficacy against lung cancer	Immunomodulation and GM regulation	Further investigation is required for clinical translation, *in vivo* efficacy validation, and safety assessment	Tian et al. (2024)
	Hemolysis assay (red blood cells)	RDLP (a homogeneous branched acidic heteropolysaccharide isolated from Rhododendron dauricum leaves, 385.4 kDa)	RDLP exhibits significant anticomplement activity	Directly inhibits excessive activation of the complement system	Confined to the biochemical level (hemolysis assay); no validation *in vitro* cell models regarding its effects on NET formation or inflammatory responses	Zhang et al. (2026)
​	HepG2 cells	WLPB-A3-c (pectic polysaccharide from Lilium); WPOP-A-c (pectic polysaccharide from Polygonatum odoratum)	The HG domains of both polysaccharides reduce H_2_O_2_-induced ROS production, increase SOD activity, and effectively protect HepG2 cells from oxidative damage	Directly scavenges free radicals; upregulates SOD activity; the HG domain (homogalacturonan region) is the primary active moiety	① Confined to *in vitro* cell models and chemical systems; no *in vivo* validation	Zhao et al. (2024)
					② Upstream signaling pathways (e.g., Nrf2, MAPK) were not investigated	
*In vivo*	Pneumonia mouse model	Tetrastigma hemsleyanum polysaccharide	① Alleviates inflammatory pathological damage	Exerts anti-inflammatory effects via the TLR4/NF-κB signaling pathway	No mention of GM, SCFAs, or intestinal barrier function	Zhou et al. (2025)
			② Reduces levels of inflammatory cytokines			
	High-fat diet (HFD)-induced obese mice (C57BL/6)	tangerine peel polysaccharide	① Alleviates hepatic steatosis and systemic inflammation	Increases intestinal acetate production by enriching acetate-producing *Bacteroides*, thereby improving metabolic disorders	① Causal relationship not validated by fecal microbiota transplantation (FMT) or germ-free animal models	Kong et al. (2026)
			② Enriches *Bacteroides* and increases intestinal acetate levels		② Target organ receptors and signaling pathways of acetate were not examined	
			③ Acetate supplementation mimics the anti-obesity effects of the polysaccharide		​	
	*In vivo* absorption study	BLLP-Cur NPs (benzoate-esterified self-assembled Lanzhou lily polysaccharide nanoparticles loaded with curcumin)	Compared with free curcumin, the nanoparticles exhibited prolonged intestinal retention time	Amphiphilic esterification modification enables self-assembly of BLLP, which contributes to prolonged intestinal retention	*In vivo* validation of combination with radiotherapy was not performed	Shi et al. (2026)
	Immunosuppressed mouse model (CTX-induced)	Flammulina velutipes residue polysaccharide (FVRP)	1 Alleviates CTX-induced intestinal injury	① Modulates GM composition	No assessment of inflammatory or fibrotic parameters in lung tissue	Yang et al. (2025)
			2 Enhances antioxidant enzyme activity and cytokine secretion	② Promotes SCFA production		
			3 Increases GM diversity	③ Enhances antioxidant enzyme activity and cytokine secretion		
			4 Increases short-chain fatty acid (SCFA) accumulation and alters metabolite levels	④ Attenuates immunosuppression and intestinal injury		
	C57BL/6J mice fed a high-fat diet (HFD) for 2 days (short-term)	Saccharina japonica polysaccharide, eight different fractions	The effects of the eight fractions on GM were evaluated. Notably, significant GM dysbiosis was already observed after 2 days of HFD feeding	Significant GM dysbiosis was already observed after 2 days of HFD feeding	Whether this polysaccharide interferes with the fundamental antitumor efficacy of radiotherapy, or exhibits synergistic or antagonistic effects with radiotherapy, was not evaluated	Ke et al. (2021)
	Tumor-bearing mouse model	PHP-1 (a homogeneous polysaccharide purified from Polygonatum cyrtonema Hua)	① Promotes *in vivo* apoptosis	Induces tumor cell apoptosis and suppresses tumor cell proliferation	No assessment of GM or gut-lung axis-related mechanisms	Gao et al. (2026)
			② Effectively inhibits tumor growth in tumor-bearing mouse models			

The above evidence suggests that LP can modulate key pathological processes in RIP. However, due to its poor oral absorption, we hypothesize that orally administered LP is unlikely to reach the lungs intact for direct action; instead, it may indirectly affect RIP progression by modulating intestinal function.

## 3 Mechanisms of LP-mediated regulation of GM and GLA immunity in RIP

### 3.1 LP modulates GM composition and homeostasis

Plant polysaccharides act as prebiotics and can be fermented by the GM into functional metabolites such as SCFAs (Keung et al., 2025; Lai et al., 2024); LP possesses this same property. The pectic polysaccharide L01-B1, isolated from Lilium lancifolium flowers, significantly increases the abundance of Bifidobacterium longum, enabling targeted regulation of probiotics through modulation of gut microecology (Li et al., 2025b). Moreover, LP substantially ameliorates GM dysbiosis by upregulating beneficial bacterial groups, including Bacteroidetes, Proteobacteria, Alistipes, and Lachnospiraceae, while downregulating Firmicutes, thereby remodeling the gut microbial community structure and its associated metabolic profiles (Li Z. et al., 2025). In addition, oral administration of LP effectively alleviates ulcerative colitis by restoring the intestinal mucosal barrier, regulating inflammatory factors, and maintaining GM homeostasis (Ji H. et al., 2025). More importantly, LP has been shown to inhibit cGAS-STING pathway activation, reduce the release of pro-inflammatory cytokines, and promote intestinal mucosal barrier repair (Wu Y. et al., 2025). Collectively, these findings suggest that LP’s regulation of the gut may serve as a critical initial step in its remote modulation of pulmonary immunity.

Extensive clinical and basic research has established that GM dysbiosis critically contributes to the onset and progression of RIP. In patients with non-small cell lung cancer (NSCLC) undergoing chemoradiotherapy, GM stability is significantly negatively correlated with RIP risk. High-risk individuals typically exhibit reduced gut microbial diversity, decreased abundance of beneficial bacteria, and metabolic imbalances (Ma et al., 2025; Xi et al., 2025). Similarly, among patients with advanced lung cancer, those who develop severe pulmonary toxicity after radiotherapy, chemotherapy, or immunotherapy show distinct GM compositions compared to mild cases, characterized by fewer mucosa-repair-associated beneficial bacteria and more pathogenic bacteria (Collado-Lledó et al., 2024; Ziaka and Exadaktylos, 2024b). Collectively, these findings suggest that gut microbial profiles may serve as valuable biomarkers for RIP risk stratification, facilitating early identification of high-risk patients and the design of personalized treatment strategies (Wu L. et al., 2025). Radiation injury has been shown to alter GM composition and diversity, leading to intestinal barrier dysfunction and bacterial translocation (Wang Q. et al., 2025). Via inter-organ communication pathways such as the GLA, these disturbances may exacerbate inflammatory responses in distant organs, including the lungs. This provides both theoretical and experimental support for GM-targeted interventions in the prevention and management of RIP.

### 3.2 GM and their metabolites: potential links to cross-organ immune modulation in RIP

Gut microbial ecology is essential for immune homeostasis. As key mediators of the gut-immune axis, the GM and their metabolites regulate immune development, activation, and homeostasis through multi-organ and multi-level mechanisms (Su et al., 2024; Wu G. et al., 2025; Zhai et al., 2025). The digestive tract, being the largest and most structurally complex peripheral immune tissue in the human body, hosts a diverse array of GM, immune cells, and gut-associated lymphoid tissue (GALT) (Mowat and Agace, 2014). Upon stimulation by bacteria or their metabolites, specific transmembrane immune cells may enter the bloodstream via lymphatic vessels and subsequently colonize distant organs such as the lungs and liver (Takeuchi et al., 2024; Wu et al., 2026). These migrating cells help modulate local immune microenvironments, thereby establishing an immunoregulatory axis connecting the gut to distant sites (Hampton and Chtanova, 2019). Evidence indicates that after recognizing microbial antigens, DCs and T cells in the intestinal lamina propria transmit antigenic signals to mesenteric lymph nodes, where immune activation occurs (Miranda-Waldetario and Curotto de Lafaille, 2024). A subset of these activated cells then enters the circulation and migrates to distant organs, including the lungs and spleen, initiating antigen-specific immune responses. This process provides a mechanistic basis for GM-mediated regulation of systemic immune diseases (Yang T. et al., 2025).

Previous studies have shown that inosine levels are significantly reduced in both the gut and lung tissues of patients with RIP (Wang S. Y. et al., 2026). As a GM-derived metabolite, decreased inosine impairs the immunoprotective function of the GLA, thereby promoting pulmonary inflammation; conversely, inosine supplementation alleviates RIP (Narikawa et al., 2025). Thoracic radiotherapy also disrupts gut microecological homeostasis, suppressing anti-inflammatory metabolites such as SCFAs while elevating pro-inflammatory metabolites, including lipopolysaccharide. Elevated lipopolysaccharide triggers a systemic inflammatory cascade, further exacerbating immune injury in the lungs (Li et al., 2020). During the RIP phase, DCs produce cytokines and growth factors that coordinate the activities of various immune cells, including macrophages, mast cells, and cytokine-induced killer cells. These activated cells subsequently release large quantities of pro-inflammatory cytokines, thereby playing a key regulatory role in RIP (Wang H. N. et al., 2025).

**FIGURE 3:**
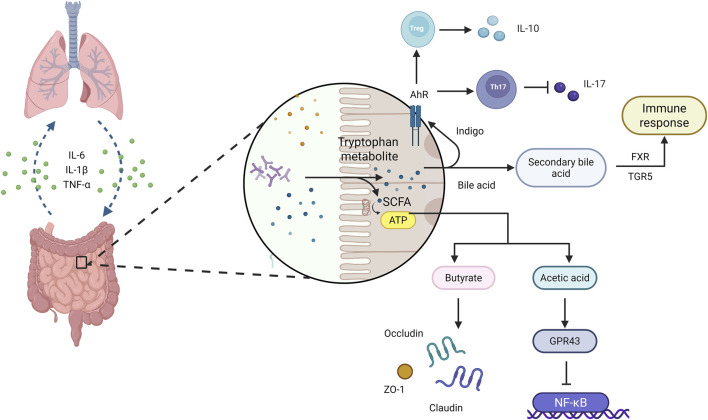
The regulation of systemic immunity by gut microbiota and their metabolites, Created in BioRender.com.

GM metabolites are crucial regulators of immune function, acting through specific targets and signaling pathways to mediate both local and systemic immune regulation (Figure 3).

#### 3.2.1 SCFAs

SCFAs, produced by GM through substrate fermentation, are essential for maintaining intestinal barrier integrity and regulating host immune responses (Niu et al., 2023). As a key energy source for colonocytes, butyrate stimulates intestinal mucosal cell proliferation and promotes the expression of tight junction proteins such as ZO-1 and occludin, thereby repairing the intestinal barrier and alleviating systemic immune responses (Peng K. et al., 2024). Acetate, via activation of GPR43, reduces the secretion of inflammatory mediators by macrophages and DCs in the intestinal lamina propria, thus relieving local inflammation (Niu et al., 2023). Furthermore, SCFAs can act directly on distant organs through the circulatory system to regulate systemic immune homeostasis (Machado et al., 2022).

In addition to classical SCFAs, 3-hydroxybutyrate (3HB) also mitigates radiation injury. Supplementation with a 3HB-producing strain of Akkermansia muciniphila alleviates radiation-induced injury (Ge et al., 2024), whereas exogenous A. muciniphila alone exacerbates acute radiation-induced intestinal injury (Wang Y. et al., 2025). Furthermore, the A. muciniphila-derived metabolite tanespimycin (17-AAG) alleviates radiation-induced intestinal injury by inhibiting the AKT/NF-κB signaling pathway and preserving intestinal barrier function (Zhang et al., 2025c). These findings add mechanistic depth to SCFA-related studies and provide a crucial foundation for developing radioprotective strategies based on microbial metabolites.

#### 3.2.2 Tryptophan metabolites

The GM degrades tryptophan into indole compounds, such as indigo and indole-3-propionic acid (Dormans et al., 2025; Jia et al., 2024; Wang J. et al., 2025). As a key immunomodulator, indigo specifically binds to the aryl hydrocarbon receptor (AhR) on the intestinal mucosal surface, precisely regulating the differentiation balance of helper T cell subsets. This mechanism suppresses IL-17 secretion by Th17 cells (Ni et al., 2024) while stimulating regulatory Tregs to produce IL-10 (Yoshimatsu et al., 2022). Furthermore, indigo modulates glucose metabolism in immune cells via the mTORC1/HIF-1α signaling pathway, effectively inhibiting aberrant immune cell activation during inflammation and thereby maintaining both local intestinal and systemic immune homeostasis (Xing et al., 2025). Oral administration of indole-3-acetic acid (IAA) alleviates radiation-induced intestinal injury by activating the AhR/IL-10/Wnt signaling axis and increasing the abundance of beneficial gut bacteria (Xie et al., 2024). Collectively, these findings expand our understanding of tryptophan metabolites and underscore the potential of targeting microbial tryptophan metabolism for radioprotective strategies.

#### 3.2.3 Bile acid metabolites

Intestinal free bile acids are converted into secondary bile acids via microbial deconjugation and dehydroxylation (Fu et al., 2025). These metabolites regulate host immune responses and glucose metabolism by modulating the farnesoid X receptor (FXR) and the G protein-coupled bile acid receptor (TGR5) (Picon et al., 2023). FXR plays a critical role in maintaining intestinal barrier integrity, thereby preventing the translocation of antigens and GM (Anderson and Gayer, 2021). Meanwhile, TGR5 activation inhibits the polarization of macrophages toward a pro-inflammatory phenotype and enhances the secretion of anti-inflammatory cytokines (Ito et al., 2021). These bile acid receptor-mediated pathways also regulate the antigen-presenting function of DCs, which in turn influences T cell activation and differentiation, ultimately enabling coordinated immune modulation of both the intestine and distant organs (Lin et al., 2025).

In conclusion, the GM may act as a central hub for host immune regulation. However, existing studies have largely focused on the local effects of gut microbial metabolites within the intestine, whereas direct evidence for their impact on the lungs remains lacking, warranting further investigation.

### 3.3 GLA bidirectional regulation in pulmonary immune homeostasis restoration and anti-inflammatory activity

GLA is central to immune homeostasis between the intestine and the lungs. Cross-organ communication along this axis involves the GM, the immune system, and their metabolites, enabling remote regulation of pulmonary immune balance and anti-inflammatory responses (Bingula et al., 2017). Within this axis, intestinal and pulmonary microbial communities interact; disruption of GM homeostasis impairs GLA stability, leading to pulmonary immune dysfunction (Wang et al., 2023). Conversely, gut-derived metabolites such as SCFAs and tryptophan metabolites reach the lungs via the bloodstream, where they activate immune cells and modulate immune responses (Chen et al., 2026; Ni et al., 2026). The bidirectional regulation of the GLA becomes particularly critical under pathological conditions such as RIP. As a direct target of radiation injury, the lungs rapidly release inflammatory signaling molecules during the acute injury phase via the lung-gut axis, which may induce GM dysbiosis and mucosal barrier damage, thereby causing secondary injury to intestinal tissue (Chen C. et al., 2025). Subsequently, the compromised gut microenvironment can transmit inflammatory signals back to the lungs through the GLA, establishing a vicious cycle of “primary lung injury-secondary intestinal injury-feedback-driven secondary lung injury,” which may accelerate RIP progression (Ziaka and Exadaktylos, 2024a) (Figure 3). Furthermore, various microorganisms in the lungs and intestine can transmit immunomodulatory signals via the GLA, promoting remodeling of the pulmonary immune microenvironment and thus playing a key role in regulating immune homeostasis in respiratory diseases (Budden et al., 2017).

The immunomodulatory functions of the GM on the host are primarily mediated by cell migration and cross-organ immune networks established by specific microbial communities (Yao et al., 2025; Zhu et al., 2025). Burrows and colleagues (Burrows et al., 2025) demonstrated that the interplay between the commensal protozoan T. mu and the bacterial GM induces chemokine receptor expression, which recruits eosinophils to form an immunoprotective barrier, thereby achieving immune regulation. Moreover, GM-derived metabolites serve as key mediators of immune function, coordinating both local and systemic immune regulation through distinct targets and signaling pathways.

Targeting the GLA has been shown to effectively alleviate lung tissue injury. For example, Houttuynia cordata polysaccharide alleviates pneumonia in mice by regulating the Treg/Th17 cell balance within the GLA and suppressing aberrant activation of the NLRP3 pathway (Li X. et al., 2025). The GM acts as a key signaling mediator in GLA function (Gopalsamy et al., 2025; Su et al., 2024) and has been reported to reduce susceptibility to fungal pneumonia through modulation of blood circulation and pulmonary metabolism (Ji J. et al., 2025). In traditional Chinese medicine, the Fuzhengjiedu formula attenuates lipopolysaccharide-induced RILI in mice by regulating amino acid metabolic pathways via the GLA (Lu et al., 2024). Additionally, forsythiaside A alleviates inflammatory injury by targeting the PPAR-γ/RXR-α complex and inhibiting the TLR4/MAPK/NF-κB and NF-κB/MLCK/MLC2 signaling cascades, further elucidating the regulatory characteristics of gut-lung crosstalk (Wang J. et al., 2024).

## 4 Conclusion and future perspectives

RIP is a common and severe complication following thoracic irradiation, yet current therapeutic options remain limited. Natural polysaccharides have attracted attention due to their multi-target activities and low toxicity. However, orally administered macro-molecular polysaccharides are poorly absorbed directly from the intestine into the bloodstream, an obstacle that opens paths for exploring their indirect mechanisms of action. The pathogenesis of RIP may be closely associated with the activation of the cGAS-STING-NLRP3 pathway induced by ionizing radiation. Nevertheless, it should be noted that the evidence linking LP-mediated GM regulation and intestinal metabolite modulation of the cGAS-STING-NLRP3 pathway remains largely indirect. Thus, the causal relationships involved require further validation using RIP models.

Based on a comprehensive analysis of available evidence, we hypothesize that oral LP alleviates RIP by remotely suppressing aberrant activation of the pulmonary cGAS-STING-NLRP3 axis via a cross-organ network involving the GM, their metabolites, and the GLA. This hypothesis links the pharmacological actions of LP and GM modulation to key molecular mechanisms of RIP, providing a new theoretical basis for clinical application of LP. However, direct evidence is lacking; the causal relationship between GM and the direct inhibition of the pulmonary cGAS-STING-NLRP3 pathway by LP metabolites has yet to be validated in RIP models. To address these gaps, future studies should employ fecal microbiota transplantation, germ-free animal models, and metabolite tracking, along with systematic investigations into structure-activity relationships of different lily varieties, intervention timing optimization, and clinical safety. In summary, as a natural polysaccharide with a favorable safety profile, LP shows potential for RIP prevention and treatment via the GLA, but further basic and clinical research is required before clinical translation.
